# Embryonic Mediators of Embryo–Uterus Communication, Implantation and Pregnancy

**DOI:** 10.1002/mrd.70137

**Published:** 2026-08-02

**Authors:** Keith E. Latham

**Affiliations:** ^1^ Department of Animal Science Michigan State University East Lansing Michigan USA

**Keywords:** assisted reproduction, embryo–uterine dialogue, preimplantation embryo development, trophectoderm, uterus

## Abstract

Successful reproduction in eutherian mammals requires intimate connections between the embryo and uterus, involving adhesion, attachment and placentation, with or without invasion of the endometrium. The complex dialogue between embryo and uterus involves numerous molecules and pathways. The study of the mechanisms driving early embryo–uterus interactions has historically been challenging, particularly discovering the embryo‐expressed molecules. Some components of the interactions have been known for decades, but many have been revealed in recent years through innovative transcriptome studies, studies of the contents and functions of extracellular particles, use of in vitro three‐dimensional organoids, genetic studies, approaches for dissecting the regulation of maternal immune system cells, and many other approaches. This review focuses on embryo‐expressed molecules that support implantation and embryo attachment, implantation and pregnancy establishment. Both classical and recent discoveries are examined, encompassing results from human, murine, livestock and comparative studies. Opportunities for further mechanistic insights are considered. Additionally, the review considers the potential for agricultural and clinical applications of the more recently identified molecular players and mechanisms for enhancing reproductive success or for providing new approaches to contraception.

## 1 Introduction

**Figure 1:**
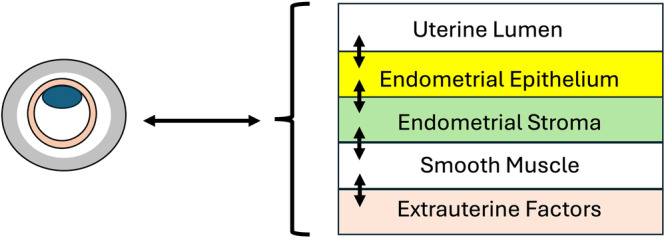
Summary of interactions that support pregnancy establishment. Signaling interactions occur between different compartments within the uterus. The embryo can modulate processes within the uterus, and in turn be influenced by factors expressed by different compartments, as appropriate to species and placentation type.

Successful reproduction in eutherian mammals requires the establishment of intimate connections between the embryonic cells and the uterus. In ungulates, the embryonic blastocyst undergoes extensive elongation and attachment to the uterine endometrial epithelium to establish intimate contacts without trophoblast (TB) invasion, leading to the formation of diffuse (epitheliochorial, e.g., pigs and horses) or cotyledonary (syndesmochorial or synepitheliochorial, e.g., ruminants) placentae. In other eutherian mammals, initial embryo attachment and adhesion to the uterine epithelium are followed by invasion into the uterine stroma leading to decidualization with chorionic villi contacting or invading the maternal vasculature. A complex dialogue enables the processes of attachment and implantation (Figure 1). Discovering the details of this complex dialogue has been a major challenge in mammalian reproductive biology due to the limited accessibility of the embryo and uterine tissue at the implantation site for study during the most critical periods of time, and the fact that detailed study at that time often requires disruption of the ongoing process. This has been especially true for studies in non‐ungulate species, in which attachment and implantation occur when the embryo and implantation site are both very small.

During the last several decades, increasingly elegant approaches have been taken to discover the essential signals that lead to attachment and implantation. These have included the use of genetically modified animal models, histological and mass‐spectroscopy analyses of placenta tissues, TB cells and TB‐like cell lines, stromal cell cultures, lipopolysaccharide‐treated cells, two‐dimensional and three‐dimensional in vitro models, in vitro time‐lapse imaging systems, air–liquid interface models, spheroid‐forming cell models, TB vesicle analyses, analyses of conditioned medium and uterine fluid, analyses of extracellular vesicles and exomeres, metabolomics, and preimplantation embryo spent culture medium analyses. Essential elements of the embryo‐maternal dialogue include the embryo signaling its presence to the mother through endocrine, exocrine, and paracrine mechanisms, establishment of a receptive state including suppression of maternal immune rejection, and early nutritive support from the uterus.

**Figure 2:**
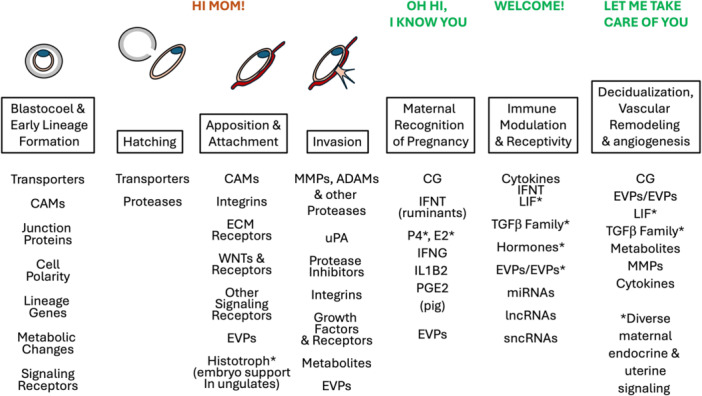
Essential steps in the embryo‐maternal dialog leading to pregnancy establishment. Embryo‐expressed genes and factors promote blastocyst formation, embryo hatching, apposition, attachment, invasion and essential uterine changes to promote successful implantation and placentation leading to sustained pregnancy. Selected maternal and uterine factors (*) are included. ADAM, a disintegrin and metalloproteinase domain gene family; CAM, cell adhesion molecule; CG, chorionic gonadotropin, ECM, extracellular matrix; EVP, extracellular vesicles and particle; IFNT, interferon tau; lncRNA, long non‐coding RNA; miRNA, microRNA; MMP, matrix metalloprotease; sncRNA, small non‐coding RNA; WNT, Wingless‐type MMTV integration site family.

The uterine processes and complex intra‐uterine regulatory mechanisms that promote adhesion, implantation and pregnancy establishment have been summarized in many excellent recent reviews. Equally important are the embryo‐expressed factors that initiate and interact with these uterine processes. However, our knowledge of embryo‐secreted factors that direct essential processes to enable pregnancy establishment (e.g., interactions with the uterine maternal immune cells) remains less complete than our knowledge of cell–cell interactions within the uterus. Because of the dynamic nature of the embryo‐maternal dialogue, understanding the remarkable complexity of the embryo‐expressed factors is vital for a complete mechanistic understanding of pregnancy establishment. A major goal of this review, therefore, is to highlight progress that has been made in this area and the knowledge gaps for further research into embryonic components of the embryo‐uterine dialogue. Moreover, understanding shared aspects of the molecular mechanisms observed across species better informs us about fundamental mechanisms of embryo attachment, implantation and pregnancy establishment, while understanding the species‐restricted features clarifies our understanding of the evolution of mammalian reproduction. Additionally, understanding the embryo side of the embryo‐maternal dialog should better inform us about the mechanisms by which environmental and genetic factors that impact the embryo also impact reproductive outcome. To fulfill these objectives, this review focuses on the embryo side of the embryo‐maternal dialog (Figure 2), addressing genes expressed by the blastocyst and early TB cells that contribute to the processes of embryo apposition, attachment and invasion, maternal recognition of pregnancy, maternal immune tolerance, and uterine changes to support pregnancy establishment (Figure 2). These gene products promote developmental changes in embryonic cellular processes and molecular components as well as the embryonic production and release of a myriad of extracellular factors that support pregnancy establishment. Some of the key connections with uterine signals are also explored. Some embryo‐derived factors have been known for many years, with some new functions and mechanisms of action discovered more recently. Other factors have been discovered more recently, including extracellular particles containing diverse cargos that participate in the embryo–uterus dialogue, microRNAs, metabolites, and other macromolecules. As these discoveries emerge, the remarkable complexity of mammalian pregnancy establishment becomes more apparent, leading to new opportunities for basic mechanistic discoveries, and the development of novel diagnostic procedures, interventions to enhance pregnancy success, contraceptive approaches, reproductive technology development, and mitigation of negative effects of adverse maternal health or environmental factors.

## 2 Morula to Blastocyst Transition and Emergence of Trophectoderm Cell Functions

The overall mission of the preimplantation embryo is to establish primary cellular lineages for embryogenesis, along with the ability to attach to the uterus and form a placenta with which to sustain further development. As embryos transition from the morula to the blastocyst stage, the outer trophectoderm (TE) cells undergo dramatic changes in gene expression profile and cellular physiology that support placentation. These extensive changes in gene and protein expression support TE cell–cell interactions to enable epithelialization, embryo cavitation and hatching, changes in TE metabolism, increased TE cell survival, and embryo attachment and invasion. They also promote subsequent activation of signaling mechanisms that underlie embryo–uterus communication as well as promoting key signaling pathways between uterine cells (Figure 2).

Epithelialization to form the TE includes the elaboration of new adhesive contacts, cell junctions, and ion transporters that collectively enable fluid‐pumping and cavitation to form the blastocyst (Ghassemifar 2003; Watson et al. 1992). The expression of these proteins is controlled by a combination of transcriptional, translational and post‐translational mechanisms (Fleming et al. 2000), with timing imposed by an intrinsic clock linked to DNA replication during early cleavage (Kidder and McLachlin 1985; Valdimarsson and Kidder 1995). Major blastocyst‐forming genes include subunits of the Na^+^/K^+^‐ATPase on the basolateral membrane of polarized cells (Goossens et al. 2007; Houghton et al. 2003; Jones et al. 1997; Watson et al. 1990), uvomorulin, catenin or CAM120/80 (Fleming et al. 1994; Watson et al. 1990), connexins (Davies et al. 1996; Houghton et al. 2002; Kidder and Winterhager 2001; Valdimarsson and Kidder 1995), desmosomal and tight junction proteins (Fleming et al. 1993, 1989; He et al. 2026), aquaporins (Barcroft et al. 2003; Offenberg et al. 2000; Watson and Barcroft 2001), and p38 MAPK to regulate cavitation and response to osmotic stress (Bell et al. 2009; Bell and Watson 2013). Na^+^/K^+^‐ATPase function is required for blastocyst formation (Madan et al. 2007) and its activity makes cavitation an energy‐intensive process. It was estimated that the Na^+^/K^+^‐ATPase consumes as much as 36% of the ATP produced in expanding bovine blastocysts and as much as 60% of the ATP produced in expanding human blastocysts (Houghton et al. 2003).

Other key TE‐expressed genes include proteases (Jousan et al. 2008; M. Ma, Zhang, et al. 2024; O'Sullivan et al. 2002, 2001; Pathak, Vani, and Seshagiri 2021; Sawada et al. 1992; Sharma et al. 2006; Vu et al. 1997; Yamazaki et al. 1994), glycoproteins, integrins and extracellular matrix (ECM) proteins (Kimber and Spanswick 2000; Turpeenniemi‐Hujanen et al. 1995), cytokines (Seshagiri et al. 2016), and growth factors and other signaling molecules (Pathak, Vani, and Seshagiri 2021). A proteomics analysis of hatching human blastocysts compared to those not yet hatching revealed enrichment for serpins, lipocalcins, and numerous proteases as well as cytoskeletal proteins (Almagor et al. 2020). Additionally, analyses of hatching‐capable human blastocysts revealed enhanced expression of cathepsin V, GATA binding protein 3 (GATA3) and human chorionic gonadotropin (CG) (Syrkasheva et al. 2017). Cathepsins are required for hamster blastocyst hatching and prostaglandin‐endoperoxide synthase 2 (PTGS2/COX2) activity, and prostaglandins promote their expression (Sen Roy and Seshagiri 2013; Seshagiri et al. 2009).

Many of the proteins that enable blastocyst formation and hatching are shared across species. However, the overall conservation of changes in gene expression going from morula to blastocyst stage is less than might be expected. A cross‐species meta‐analysis of embryo transcriptomes for five mammalian species during the morula‐to‐blastocyst transition revealed that, within any single species, there are large numbers of genes that are up‐ or down‐regulated during this transition, or that are differentially expressed in the TE as compared to inner cell mass (ICM) cells (Schall and Latham 2021). However, the number of genes displaying comparable changes across all five species was quite limited (Schall and Latham 2021). Despite this, there was considerable conservation in cellular pathways and biological functions associated with the morula‐to‐blastocyst transition, indicating that different species regulate cohorts of genes differently to achieve comparable endpoints (Schall and Latham 2021). The most highly shared biological functions for this transition included increases of metabolism of membrane lipid derivatives, fatty acid metabolism, endocytosis, transport of molecules, invasion of cells, organization of cytoplasm, and organization of cytoskeleton, reduction of organismal death, and additional effects (direction not consistently predicted) on cell survival and viability, cell movement, cell contact, and migration of cells (Schall and Latham 2021). The most highly shared affected biological functions for TE‐enriched genes included cell survival, size of embryo, and numerous entries related to cell migration, cell invasion, and cell–cell contact (Schall and Latham 2021). The TE‐expressed genes described above, together with this transcriptome meta‐analysis, are thus suggestive of shared changes in gene expression that support the essential properties of epithelialization, cavitation, and hatching as a prelude to endometrium adhesion and species‐appropriate cell attachment and invasiveness.

## 3 Blastocyst‐Expressed Receptors That Support Attachment and Implantation

As the blastocyst enters the uterus a complex dialogue occurs wherein uterine factors promote blastocyst health and activation to an implantation‐competent state (Geisert et al. 2012; He et al. 2019; Massimiani et al. 2019; C. Wang and Dey 2006) (Figure 2). Blastocyst‐derived factors promote immune tolerance, and the TE establishes an intimate connection with the uterus either through attachment and formation of specialized structures (e.g., placentomes in ruminants, or areolae in pigs) or attachment followed by invasion and implantation. Subsequent placentation establishes a vascular supply to nourish and sustain the embryo. In mice, implantation occurs within specialized implantation chambers or crypts that form where the embryo attaches (Yuan et al. 2018; Aikawa et al. 2024). Additionally, structures at the surfaces of endometrial luminal epithelial cells called pinopodes are proposed to contribute to embryo‐uterine interactions in diverse species, appearing at sites of interaction during the window of implantation (Quinn et al. 2020).

**Figure 3:**
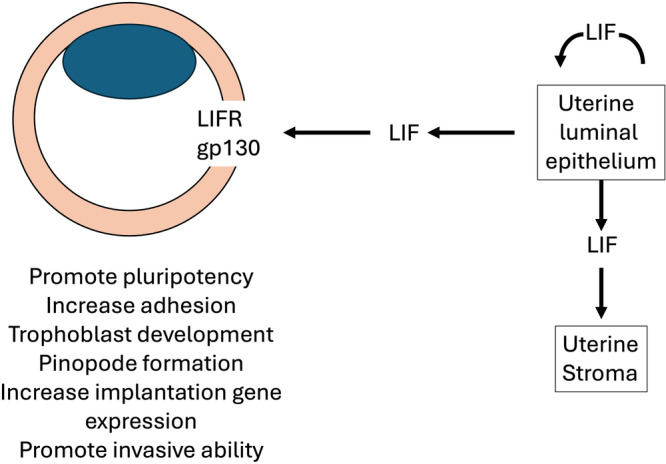
Summary of leukemia inhibitory factor (LIF) actions in the uterus and embryo in support of pregnancy establishment. LIF from the uterus can activate embryonic LIF receptor (LIFR and glycoprotein gp130) to support early processes in the embryo, such as establishment of pluripotency. Further actions are exerted on TB cells. Within the uterus, LIF expression and signaling mediates essential processes within the epithelium and between the epithelium and stroma.

Some uterine factors support the embryo via embryo‐expressed receptors. One example is uterine‐derived leukemia inhibitory factor (LIF) (Kauma and Matt 1995; Salleh and Giribabu 2014; Stewart et al. 1992), which affects both embryo and uterine functions (Figure 3). LIF promotes pluripotency in embryos of diverse species including mice, humans and sheep (Vogiagis and Salamonsen 1999). LIF enhances bovine and sheep embryo development in vitro (Bao et al. 2014; Campanile et al. 2021; Neira et al. 2010). In mice, LIF antibody reduces embryo implantation and LIF enhances blastocyst outgrowth in vitro (Cai et al. 2000). Conditional knockout of forkhead box A2 (*Foxa2*) in mice leads to severe infertility and a block to blastocyst implantation due to a deficiency in uterine glands and their production of LIF to support the embryo. LIF also promotes uterine endometrial receptivity, embryo chamber formation, and embryo attachment, and together with other factors promotes uterine decidualization (Aikawa et al. 2024; Dhakal et al. 2021; Jeong et al. 2010; Kelleher et al. 2017; Salleh and Giribabu 2014). Endometrial LIF deficiency in humans may contribute to infertility (Vogiagis and Salamonsen 1999). Importantly, although some aspects of LIF expression and function in the uterus and its effects on the embryo are similar across species, the temporal expression patterns of LIF in the endometrium and its specific functions during implantation vary across species due to differences in specific endocrine regulation of LIF gene expression, differences in cytokine regulation and involvement, and differences in placentation (Campanile et al. 2021; Vogiagis and Salamonsen 1999).

**Figure 4:**
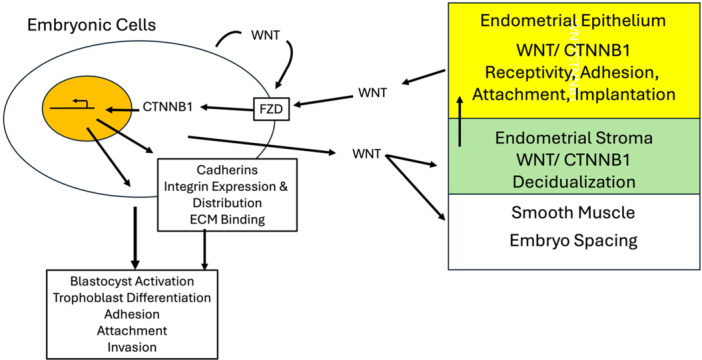
Summary of WNT and catenin B1 (CTNNB1) signaling and functions in pregnancy establishment. The embryo produces WNTs, which can act in an autocrine fashion. WNTs from the uterus act on the early embryo via the frizzled receptor (FZD) to promote expression of cadherins, integrins and ECM molecules, which serve a range of functions, including blastocyst activation, preparing the embryo for the initial interaction with the luminal epithelium, and promoting TB differentiation. Embryonic WNTs in turn affect the uterus, for example by promoting decidualization and controlling embryo spacing. Within the uterus, WNT signaling from the stroma affects epithelial receptivity and adhesiveness and success of embryo implantation.

The WNT (Wingless‐type MMTV integration site family) signaling pathway plays a vital role in embryo attachment and invasion, not only within the endometrial epithelium, but also within the embryo (Figure 4). WNT signaling is a basis for important embryo‐uterine communication during attachment and implantation, with uterine WNTs modifying the endometrial epithelium to enable attachment, uterine WNTs promoting blastocyst competence for implantation and TB proliferation, and embryonic WNTs eliciting uterine changes at the implantation site to promote receptivity and embryo attachment (Chen et al. 2009; Lou et al. 2023; Qin et al. 2025).

**Figure 5:**
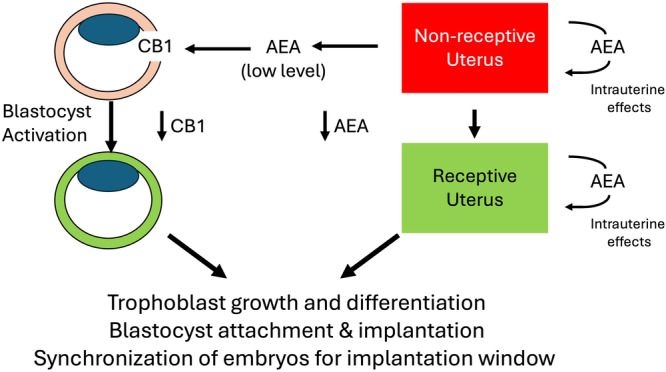
Summary of identified endocannabinoid signaling functions in the embryo and uterus to support pregnancy establishment. Uterine anandamide (AEA) at the appropriate level exerts positive effects on the early embryo via signaling through the embryonic cannabinoid (CNR1/CB1) receptor. Upon blastocyst activation and signaling to the uterus to transition to a receptive state, embryonic CB1 is down‐regulated and the uterine AEA level diminishes. These changes contribute to TB growth and differentiation, attachment, implantation and appropriate synchronization of embryo development relative to the temporal window of receptivity.

The endocannabinoid system mediates diverse functions in human reproduction, including roles in oviductal embryo transport, embryo implantation, modulation of immune cell function, effects on spiral artery remodeling and control of the timing of receptivity for implantation (Pařízek et al. 2023) (Figure 5). Uteri produce two endogenous cannabinoids (anandamide [AEA] and 2‐arachidonoylglycerol [2‐AG]) as ligands that can interact with the cannabinoid receptor 1 (CNR1/CB1). Much of the research on cannabinoid effects has examined AEA regulation and has used AEA experimentally. CB1 function can promote blastocyst attachment and migration (Kim et al. 2021; Liu et al. 2002; Schmid et al. 1997). Cannabinoid signaling within the uterus contributes to decidualization and other processes during pregnancy (Y. Li, Dewar, et al. 2020; Pařízek et al. 2023). Uterine‐derived AEA promotes embryo development at low concentrations but can be detrimental at higher concentrations. Embryos lacking cannabinoid receptors display delayed, asynchronous development, which though compatible with implantation may be disruptive of synchronization with the window of implantation (Correa et al. 2016; Paria et al. 2001; Sun and Dey 2009). However, excess cannabinoid stimulation can inhibit embryogenesis, TB development, hatching and implantation. Elevated maternal AEA is associated with uterine non‐receptivity. Embryonic cannabinoid receptors are downregulated in activated blastocysts (Paria et al. 2001; Pařízek et al. 2023). Mouse blastocysts release a lipid that can promote the reduction of uterine AEA (Schmid et al. 1997). In combination, downregulation of AEA at the implantation site and reduced expression of embryonic CB1 receptor promote embryo implantation (Pařízek et al. 2023). Thus, achieving the optimum level of CB1 stimulation in the embryo in conjunction with timely modulation of endocannabinoid levels at the implantation site contributes to synchronization between embryo development and uterine receptivity and promotes successful implantation (Paria et al. 2001).

Pig blastocysts express prostaglandin F (PGF) receptors (Kaczynski et al. 2018). Although peripheral PGF functions as a luteolytic signal (Kaczynski et al. 2018), PGF within the uterine lumen may promote attachment by promoting endometrial expression of PTGFR (PGF2α receptor), vascular endothelial growth factor A (VEGFA), matrix metalloprotease 9 (MMP9), and transforming growth factor beta 3 (TGFβ3), by promoting TB proliferation and adhesion to the ECM, and by promoting angiogenesis within the endometrium at the implantation site (Kaczynski et al. 2018; Kaczynski et al. 2020; Kaczynski et al. 2016).

Many growth factors and cytokines that can affect embryos are produced either by the maternal reproductive tract or by the embryo itself (Adamson 1993; Bazer and Johnson 2014; Massimiani et al. 2019; McDonald et al. 2025; Minas et al. 2005; Pampfer et al. 1994; Schultz and Heyner 1993; Stewart et al. 1992). Some embryo signals to the uterus may elicit reciprocal signaling in the form of growth factors and cytokines, and uterine factors stimulate ICM survival or expansion, or regulate TE cell proliferation, adhesion to the endometrium, and invasion.

The rapid elongation of ungulate blastocysts creates a very high energy demand (Johnson, Seo, et al. 2023). Ungulate uteri secrete a complex mixture of nutrients and other molecules that is collectively termed ‘histotroph’ to support embryo elongation and growth (Liu et al. 2025; Paudel et al. 2025). Histotroph comprises a complex mixture of hexose sugars, essential and non‐essential amino acids, proteins, lipids, ions, and growth factors and other components (Bazer et al. 2015; Johnson, Bazer, et al. 2023). A key component of histotroph is osteopontin, an ECM molecule that plays crucial roles in pregnancy establishment across species (Johnson et al. 2014). The embryos of diverse species also express a wide range of transporters for uptake of histotroph components, including many sugar and amino acid transporters (Bazer et al. 2015; Johnson et al. 2023). Some of the amino acids serve not only nutritive roles but also provide the embryo with essential capacities for critical signaling pathways, such as leucine and arginine for signaling through TOR and nitric oxide, and their transporters (e.g., SLC7A1) are essential for development and pregnancy (Bazer et al. 2015; Johnson, Bazer, et al. 2023). Histotroph is also produced and contributes to pregnancy in other mammals, including mice and humans, and the yolk sac can uptake these nutrients via many expressed transporters (Burton et al. 2020; Cindrova‐Davies et al. 2017). Interestingly, in ruminants the embryo indirectly stimulates histotroph production via a progesterone‐mediated pathway, and in turn must express the necessary receptors and transporters for histotroph uptake.

The embryonic expression of genes participating in attachment, implantation and invasion is regulated in part by extensive endocrine and paracrine signals provided by the uterus (Aikawa et al. 2022; Akaeda et al. 2024; J. Cheng, Sha, et al. 2023; Jafari‐Gharabaghlou et al. 2021; Logsdon et al. 2023; Tepekoy et al. 2015; Wetendorf and DeMayo 2012; Williams et al. 2024; H. M. Wu et al. 2024). Growth factors promote TB differentiation, proliferation, migration, and invasion (Hu et al. 2024; Lan et al. 2024; Lou et al. 2023; Tang et al. 2022). Other uterine factors promote and regulate invasion by regulating and augmenting embryonic proteases and their activities, by regulating cell adhesion molecules, by modulating the uterine environment (e.g., decidualization, endometrial cell polarity, endometrial integrity), by facilitating embryo hatching, attachment, implantation, invasion and survival, and by modulating embryonic signaling to the uterus (Hiraoka et al. 2025; Jones et al. 2002; Jones et al. 2006; Lai et al. 2022; Lan et al. 2024; Menkhorst et al. 2010; O'Sullivan et al. 2004; Oozono et al. 2008; Pathak, Vani, Sharma, et al. 2021; Sharma et al. 2013, 2011, 2006, 2016; Shmygol and Brosens 2021; Stoikos et al. 2008; Vasquez et al. 2018; Visser et al. 2018; Z. Yang, Yan, et al. 2025; You et al. 2021; Zhou et al. 2024). More recently, uterine extracellular vesicles and particles (EVPs) have been discovered, with cargos regulating the uterus itself as well the embryo (Bang et al. 2025; H. M. Wu et al. 2024).

The contribution of the maternal immune system to implantation and pregnancy is substantial, regulating both the uterine structure and activities and gene expression in embryonic cells. The immune cells present within the uterus have unique phenotypes and produce crucial cytokines required for implantation (Zenclussen and Hämmerling 2015). The maternal innate immune system in the uterus contributes not only to uterine tissue remodeling, spiral artery remodeling, and immune suppression, but also the production of diverse factors that affect TB functions; ablation of uterine immune cells or their regulators can prevent pregnancy establishment (Burns et al. 2026; Kanter et al. 2021; Kareus et al. 2026; Qiu et al. 2025; C. Wang et al. 2026; Yull et al. 2023; Zenclussen and Hammerling 2015). The emerging discoveries of maternal immune cell actions within the uterus that modulate embryonic cell properties are providing unprecedented new knowledge about crucial cell–cell interactions within the uterus that enable embryo implantation.

## 4 Blastocyst Expressed Cell Adhesion Genes Required for Attachment, Adhesion and Interaction With the Basement Membrane

The blastocyst must initially contact and adhere to the endometrial luminal epithelium. An adhesion cascade has been surmised in diverse species, wherein interactions between TE cells and the luminal epithelium progress from close initial apposition to adhesion between the apical surfaces of TE cells and luminal epithelial cells (Kimber and Spanswick 2000). The adhesion cascade in ungulates is initiated and regulated by interferons and other cytokines (Johnson et al. 2025; Spencer et al. 2004). Changes in the expression of adhesion molecules at the luminal epithelial cell surfaces occur as well as downmodulation of MUC1, which otherwise masks adhesion factors (Kimber and Spanswick 2000). The details of the adhesion cascade vary according to species and type of placentation (Kimber and Spanswick 2000). Adhesion begins with interactions between multiple carbohydrates and lectins. In hemochorial species the embryo invades through the basement membrane and then contacts and invades the ECM, allowing further cytotrophoblast migration and proliferation. In epitheliochorial or synepitheliochorial species, osteopontin serves as a major ECM molecule supporting and strengthening cell–cell interactions between the embryo and the uterine epithelium (Johnson et al. 2014). Changes in the uterine ECM during implantation can modify TB integrin gene expression (Armant 2005). In addition to allowing for physical attachment and invasion, the interactions of the TB cells with the ECM bring them into proximity of immune system cells for essential paracrine interactions. These interactions also initiate and modulate within the TB cells the expression and activation of other factors involved in invasion.

The initial interaction between embryo and the luminal epithelium and subsequent interactions are mediated by a variety of cell–cell and cell‐ECM adhesion molecules. Multiple mechanisms contribute to the downregulation of MUC1 on the endometrium, creating an opportunity for embryo apposition and adhesion (Inyawilert et al. 2014, 2015; Johnson et al. 2001; X. Wang, Li, et al. 2025). Embryo‐expressed trophinin may contribute to initial human blastocyst apposition, adhesion and subsequent cell–cell interactions during invasion, and activate TB cell proliferation (Fukuda and Sugihara 2008). Following initial apposition and attachment, the invading embryo in hemochorial species interacts with the uterine ECM. Fibronectin and laminin, two components of the endometrial basement membrane, can promote in vitro mouse blastocyst attachment and outgrowth (Armant et al. 1986), and are also seen in association with TB cells in human placentae (Earl et al. 1990; Korhonen and Virtanen 1997). Uterine entactin facilitates mouse and human TB adhesion to ECM (Yang et al. 1996; Yelian et al. 1993). Embryonic heparan sulfate proteoglycan perlecan is associated with attachment competence (Carson et al. 1993). Collagen in the basement membrane can promote ECM attachment, uterine receptivity and embryo invasion (Griffiths et al. 2019; Kuo et al. 2018). Other ECM components are observed in the endometrial basement membrane and stroma for the embryo to interact with during invasion (Maquoi et al. 1997; Stumm and Zorn 2007). Beta 1‐6 branched oligosaccharides may modulate ECM attachment allowing TB invasion (Yagel et al. 1990). Embryonic and uterine galectins may play multiple roles in implantation and embryo‐maternal interactions, including ECM interaction, uterine receptivity, TE lineage differentiation, fusion, attachment and invasion (Legner et al. 2024; Maquoi et al. 1997; Oravecz et al. 2025). Human cytotrophoblasts express the cell adhesion molecule e‐cadherin (Fisher et al. 1989). Platelet‐endothelial cell adhesion molecule‐1 (PECAM) and intercellular adhesion molecule (ICAM) in the endometrium may also facilitate TB adhesion and placentation (Coukos et al. 1998; Lai et al. 2022).

Osteopontin has emerged as a conserved ECM component that promotes embryo adhesion as an integrin receptor, particularly in ungulates, but also in hemochorial species such as mice and humans (Garlow et al. 2002; Johnson et al. 2014; Joyce et al. 2005). Osteopontin is released by uterine glands in ungulates and produced by uterine natural killer cells in mice and by the decidua in humans, and it binds to the integrins on apical surfaces of both the luminal epithelium and TE cells, thereby promoting adhesion (Johnson et al. 2014). Embryonic integrins binding osteopontin have been identified for several species, including αvβ6 in pigs and sheep, and αvβ3 in humans and rodents (Erikson et al. 2009; Johnson et al. 2014). A role for αvβ6 integrin in binding osteopontin in TE cells may be related to its best‐known roles in activating TGFβ1 signaling (Koivisto et al. 2018), which plays a role in pig placentation (Massuto et al. 2010). Osteopontin expression becomes increased at large focal adhesion sites, and there are bi‐directional responses wherein the embryo promotes osteopontin expression in the uterus, osteopontin activates key signaling pathways in TE cells to activate adhesion, migration and cytoskeletal changes, and the TE cells in turn increase osteopontin expression at the sites of uterine contact (Johnson et al. 2014). Further studies of how osteopontin‐integrin interactions are integrated with other adhesive mechanisms and signaling processes across different species should provide further insight into the complex embryo‐uterine dialog.

The interaction of embryo‐expressed integrins with the endometrium is critical for adhesion and invasion, mediating both physical interactions and complex signaling cascades that enhance these processes. A range of integrins have been detected in blastocysts and TB cells of different species, or predicted based on transcriptomic data, and specific integrins allowing interaction with specific ECM molecules have been identified (Bazer and Johnson 2024; Damsky et al. 1992; Goossens et al. 2009; Johnson et al. 2001; Johnson, Burghardt, et al. 2023; Kimber and Spanswick 2000; Klaffky et al. 2001; Lan et al. 2024; Malak and Bell 1994; Merviel et al. 2001; Velho et al. 2019; J. E. Wu and Santoro 1994; Yang et al. 1996). (Table 1). Also expressed are factors that regulate integrin function (Lou et al. 2023; Montanez et al. 2008). Binding to the ECM via integrin heterodimers elicits signaling events and protein redistributions within the TB cells to further regulate adhesion and invasion (Lan et al. 2024; J. Wang et al. 2002, 2007). Comparative data obtained for integrin expression across species indicate differences; however, such expression data may be incomplete, and, overall, there is considerable conservation, even across hemochorial, epitheliochorial and synepitheliochorial species (Table 1). The emergence of powerful new data sets such as single cell RNA sequencing combined with spatial information for different species may provide important new insights into integrin expression, regulation and function during blastocyst attachment and invasion, and the roles of integrin‐ECM interactions during pregnancy establishment across species.

**Table 1:** Blastocyst‐expressed integrin heterodimers observed or potentially expressed for mediating embryo–ECM interactions in different species.

Integrin	Major potential ECM targets	Mouse	Human	Sheep	Pig	Cow
α1β1	CO, LM					
α2β1	CO, LM	TRUE				TRUE
α3β1	CO, LM, FN		TRUE			TRUE
α4β1	FN			TRUE	TRUE	
αvβ1	CO, FN, VN, OPN		TRUE	TRUE	TRUE	TRUE
αvβ3	LM, FN, VN, TN, OPN	TRUE	TRUE	TRUE	TRUE	
αvβ5	FN, VN, OPN		TRUE	TRUE	TRUE	TRUE
αvβ6	FN, OPN			TRUE	TRUE	
α5β1	FN, OPN	TRUE		TRUE	TRUE	TRUE
α6β1	LM	TRUE	TRUE			
α6β4	LM		TRUE			
α7β1	LM	TRUE	TRUE			
α8β1	FN, VN, OPN, TN					TRUE
αIIβ3	FN, VN	TRUE				

*Source:* Data from Merviel et al. (2001), Kimber and Spanswick (2000), Johnson et al. (2001, 2023), Bazer and Johnson (2024), J. E. Wu and Santoro (1994), Klaffky et al. (2001), Goossens et al. (2009), Velho et al. (2019).

Abbreviations: CO, collagen; FN, fibronectin; LM, laminin; OPN, osteopontin, TN, tenascin; VN, vitronectin.

## 5 Blastocyst‐Secreted Factors Supporting Invasion, Growth and Survival

The blastocyst produces numerous factors to facilitate implantation and embryo survival in the uterus. These factors promote a variety of processes such as hatching from the zona pellucida, invasion, endocrine and paracrine communication, maternal recognition of pregnancy and immune suppression. They also regulate other factors that affect embryo growth and survival. A myriad of such factors has been identified through a variety of approaches, including extensive proteomic analyses of secreted proteins in several species (human, cow, sheep, mouse, and cat) as well as studies of exomeres and extracellular vesicle components (miRNAs, mRNAs, sncRNAs, etc.), metabolites, and the expression profiles of a number of other candidate classes of molecules, such as endocrine and paracrine factors. The identification of these factors and how their expression varies under different conditions has provided insight into important developmental mechanisms and yielded potential markers of embryo quality.

### 5.1 Proteases and Protease Inhibitors

Blastocysts produce and secrete proteolytic enzymes that perform multiple key functions, including hatching, invasion through the basement membrane, ECM degradation, modulation of extracellular signaling and receptor activation, modulation of uterine cell activities, activation of regulatory molecules, and suppression of cytokine activity and immune attack. Prominent categories of proteases involved in these essential proteolytic processes include matrix metalloproteases (MMPs), proteases known as ADAMs (a disintegrin and metalloproteinase domain), serine proteases including plasminogen activators (Vu et al. 1997), high temperature requirement factor A1 and A4 (HTRA1, HTRA4), membrane‐bound dipeptidyl peptidase IV (DPPIV), the transmembrane serine protease hepsin, and cysteine proteases including cathepsins.

The MMPs comprise a family of 17 different endopeptidases, most of which degrade basement membrane and ECM components such as collagen, fibronectin, laminin, and elastin (Piperigkou et al. 2025; Zhu et al. 2012). MMPs are classified into sub‐types based on substrates and on whether they are secreted or membrane‐associated (Gualdoni et al. 2022). Multiple MMPs are expressed in embryos and TB cells and serve roles in TB invasion and maternal tissue remodeling (Brenner et al. 1989; Gualdoni et al. 2022; Zhu et al. 2012). Chief among these are MMP2 and MMP9, particularly at early stages of implantation (Fang et al. 2021; Fu et al. 2017; Gualdoni et al. 2022; Legner et al. 2024; Shimonovitz et al. 1994; L. Wang, Yu, et al. 2015; Zhang et al. 2020; Zhu et al. 2012). The timing and levels of expression and activity for individual MMPs varies with species and placenta type (Gualdoni et al. 2022). Other MMPs contribute to TB invasion as well as spiral artery and tissue remodeling (Gualdoni et al. 2022; Luo et al. 2015; Tinsley et al. 2025; Zhu et al. 2012). MMP activity is regulated by tissue inhibitors of metalloproteases (TIMPs) expressed by embryos and TB cells as well as in the uterus (Alexander et al. 1996; Brenner et al. 1989; Graham and Lala 1992; Reponen et al. 1995; Whiteside, Jackson, et al. 2001; Xu et al. 2001; Zhang et al. 2003). Additional regulation is provided by plasminogen activators (PAs), such as urokinase‐type plasminogen activator (uPA) expressed in the blastocyst (Sappino et al. 1989), and PA surface receptors. The PAs activate MMPs and are regulated by PA inhibitors (PAIs) and PAI regulators expressed in the uterus and in TB cells (Aflalo et al. 2005; Carroll et al. 1993; Feinberg et al. 1989; Feng et al. 2001; Herz et al. 1992; Sappino et al. 1989; Zhang et al. 1994). Numerous other regulators and diverse signaling mechanisms operating at multiple levels to control MMP activity have been identified, including regulation by the ECM, membrane‐bound proteases, paracrine and endocrine factors, and cytokines (Bischof and Campana 2000; J. Chen, Song, et al. 2023; J. C. Cheng, Meng, et al. 2023; Feng et al. 2001; Fu et al. 2017; Fujiwara et al. 2005; Furmento et al. 2014; Gualdoni et al. 2022; Jia et al. 2014; Lash et al. 2006; Li et al. 2014; Liu et al. 2020; Minas et al. 2005; Z. Wu, Fang, et al. 2022; Xu et al. 2001; Yang et al. 2014; Zhu et al. 2012). A complex set of regulatory mechanisms thus controls the timing, protein substrate, and degree of MMP activity and ultimately TB invasiveness. Dysregulation of MMP activity may lead to defects in invasion, placenta defects, preeclampsia, and gestational TB disease (Rahat et al. 2016; Uchide et al. 2016; Zhu et al. 2014; Zhu et al. 2012). Interestingly, MMPs are also expressed in preimplantation cow, sheep and pig embryos as well as mouse (Whiteside, Kan, et al. 2001). In cows, with cotyledonary synepitheliochorial placentae, an early pre‐blastocyst pattern of MMP9 and uPA expression was interpreted to indicate potential roles distinct from those in species with hemochorial placentae, perhaps in regulating growth factor bioavailability or ECM remodeling (Whiteside, Kan, et al. 2001).

The ADAMs comprise another family of proteases expressed in TB cells that target and regulate other proteins such as growth factors, cytokines, receptors and their ligands (Pollheimer et al. 2014; Takahashi et al. 2014). Trophoblast cells express ADAMs in a developmentally regulated manner that may facilitate invasion (Aghababaei et al. 2014; Li et al. 2021; Pollheimer et al. 2014; Takahashi et al. 2014).

Cysteine proteases also play key roles in hatching and TB invasion. In hamsters, interleukin 1 beta (IL1B) promotes embryonic cathepsin ‐L and cathepsin‐B gene expression and promotes hatching, and interleukin 1 receptor antagonist (IL1RA) inhibits these processes (Pathak, Vani, and Seshagiri 2021). Cathepsins‐L, ‐B, and ‐P promote hamster embryo hatching by degrading the zona pellucida. (Sen Roy and Seshagiri 2013). Cyclooxygenase‐2 (PTGS2/COX2) is critical for hatching and promotes cathepsin expression in hamster blastocysts (Sen Roy and Seshagiri 2013). Cathepsins are also expressed in human (Divya et al. 2002) and mouse (Afonso et al. 1997, 1999; Nakajima et al. 2000) TB cells and are required for invasion (Afonso et al. 1997). Cathepsin activity is regulated by maternal cathepsin inhibitor cystatin C as well as maternal cathepsin S (Afonso et al. 1997). Interestingly, blastocysts also express cystatin C and F, which are believed to regulate the depth of their invasion as well as inhibiting maternal cathepsin‐S (Afonso et al. 1997). Cathepsin‐L is secreted by high‐quality bovine blastocysts and facilitates bovine blastocyst hatching (Raes et al. 2023). In contrast to the above roles of cathepsins in hatching and invasion, embryonic cathepsin‐D expression negatively correlates with bovine embryo quality, is induced in heat shocked bovine embryos, and may activate caspases driving apoptosis (Balboula et al. 2013). Additionally, inhibiting cathepsin B can enhance bovine embryo development (Balboula et al. 2013).

Other known or potential protease inhibitory functions are expressed in embryos or placental TB cells, including Kunitz domain‐containing serine protease inhibitors, a micro‐RNA inhibitor of low‐density lipoprotein receptor related protein 6, and alpha‐1 antitrypsin (A1AT) (Duffy et al. 1997; Kohama et al. 2012; Ni et al. 2021; Yoshida et al. 2022; Zhang et al. 1998). However, despite the presence of certain protein domains, considerable diversity in protein sequences and inhibitory properties (e.g., Kunitz family proteins) have been seen, raising the possibility that some of these proteins play roles other than protease inhibition (Chakrabarty et al. 2006; Duffy et al. 1997; MacLean et al. 2003; MacLean et al. 2004). The uterus also expresses protease inhibitors under the control of hormones and cytokines (Bischof et al. 2000; Jing et al. 2022). These protease inhibitors, along with other spatial and temporal regulatory mechanisms controlling protease expression and activities, provide maternal control of TB invasiveness, preventing excessive invasion in hemochorial species and limiting invasiveness in epitheliochorial or synepitheliochorial modes of placentation (Bazer and Roberts 1983; Lala and Graham 1990; Roberts and Bazer 1988; Salamonsen 1999; Woessner 1996). An imbalance between protease and protease inhibitor activities is associated with trophoblastic diseases (Chen and Khalil 2017; Rahat et al. 2016).

### 5.2 Hormones and Cytokines

**Figure 6:**
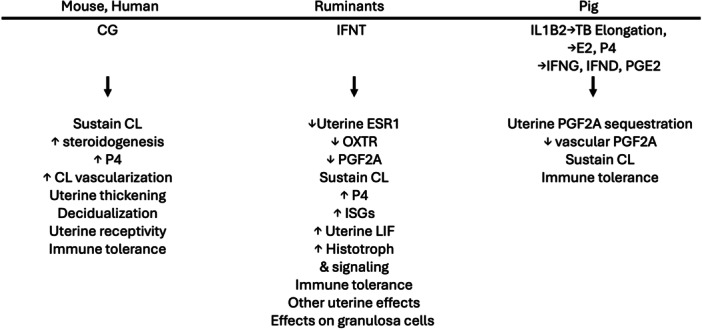
Summary of mechanisms of maternal recognition of pregnancy (MRP) in different species. MRP is mediated by chorionic gonadotropin (CG) in mice and humans, by interferon tau (IFNT) in cattle and by a servomechanism in pigs involving sequential actions of key components including the production of estradiol (E2) and progesterone (P4) by the embryo. These factors elicit essential uterine responses, including sustaining the corpus luteum (CL), maternal immune tolerance, and establishing uterine receptivity. IFND, interferon D; IFNG, interferon gamma; IL1B2, interleukin 1 beta 2; ISG, interferon stimulated gene; LIF, leukemia inhibitory factor; OXTR, oxytocin receptor; PGE2, prostaglandin E2; PGF2A, prostaglandin F2α; PGF2A, prostaglandin F2α; TB, trophoblast.

Blastocysts and TB cells within the placenta secrete multiple peptide hormones that are essential for the crucial processes of maternal recognition of the conceptus (Figure 6), metabolic regulation, and pregnancy maintenance (Ahmadi et al. 2025). Blastocysts secrete chorionic gonadotropin (CG), which is comprised of two subunits and is present in four distinct forms differing in post‐translational modifications (d'Hauterive et al. 2022). The timing and amount of embryonic expression of CG types may indicate the likelihood of successful implantation (Butler et al. 2013). CG acts in an endocrine fashion to sustain the corpus luteum thereby promoting progesterone production, which promotes thickening and maintenance of the uterus, and exerting other effects on the mother (Cole 2010; Gridelet et al. 2020; Hanson et al. 1971). CG promotes cell fusion to create syncitiotrophoblast formation (Cole 2010). Additional CG functions within the uterus or uterine cells in culture include promoting decidualization, promoting MMP expression and suppressing TIMP expression in decidualized endometrial stromal cells, repressing interleukin‐6 (IL6) expression and inducing LIF in endometrial epithelial cells, inhibiting uterine contractility, promoting angiogenesis and vasculogenesis (observed in vascular cells in vitro, but possibly balanced by anti‐angiogenic factors from endometrial stroma cells), promoting uterine receptivity and immune tolerance, eliciting metabolic changes, and initiating diverse signaling pathways (Berndt et al. 2013; Fluhr et al. 2008; Gridelet et al. 2020; Makrigiannakis et al. 2017; Perrier d'Hauterive et al. 2004; Riboldi et al. 2020; Voros et al. 2025). It can also protect endometrial stromal cells from stress‐induced apoptosis in vitro, and its hyperglycosylated form is positively associated with implantation in humans (d'Hauterive et al. 2022; Kajihara et al. 2011; Sasaki et al. 2008). A recent review highlighted in detail the many effects of hCG within the uterus, and the potential value of hCG as an intervention tool for use in enhancing human in vitro fertilization outcomes (Voros et al. 2025).

Ruminant blastocysts produce a unique interferon called interferon tau (IFNT), originally designated as trophoblast protein 1 (Bazer et al. 2025, 1991; Bazer and Thatcher 2017; Helmer, Gross, et al. 1989; Helmer, Hansen, et al. 1989; Roberts et al. 1990; Thatcher et al. 1989, 1995; Vallet et al. 1989). Extensive research on IFNT over several decades uncovered a broad range of functions in ruminant pregnancy establishment, including maternal recognition of pregnancy (Batool et al. 2025) (Figure 6). Embryonic IFNT expression may commence in response to endogenous progesterone‐induced retrovirus production by the uterus and signaling via toll receptors (Bazer et al. 2025). There is variability in the timing and mechanisms of action of IFNT in different ruminants. In sheep, IFNT inhibits uterine expression of estrogen receptor ESR1, blocking expression of oxytocin receptor (OXTR) and attendant oxytocin‐stimulated uterine production of prostaglandin F2 alpha, thereby preventing corpus luteum regression and promoting progesterone production to maintain pregnancy (Bazer et al. 2025, 1997, 1991; Bazer and Thatcher 2017; Johnson et al. 2024; Spencer et al. 2004). Interestingly, progesterone receptors are downregulated in uterine luminal and glandular epithelial cells responsible for essential progesterone‐mediated functions, and it has been suggested that progesterone‐responsive stromal cells release progestamedins to promote these essential functions (Bazer et al. 2008; Johnson et al. 2018). IFNT activates Janus kinase/signal transducer and activator of transcription (JAK/STAT) signaling, and other transcriptional regulators to activate interferon‐stimulated genes, suppress ESR1 and OXTR, and regulate diverse other functions in the uterus (Bazer et al. 2025, 1997; Johnson et al. 2024). In cattle, IFNT also acts via JAK/STAT signaling to suppress PGF2α production (Thatcher et al. 2001). IFNT has extensive effects on the uterine endometrium (Mathew et al. 2019). Secretion commences on day 8 or 9 in sheep, and day 10 in cattle (Flint et al. 1994). IFNT in the endometrium modulates PI3K/AKT/β‐catenin/FoxO1 signaling and other pathways to regulate inflammation and maternal immune tolerance, and inhibit viral infection (Bazer et al. 2008; Feng et al. 2025; Jiang et al. 2024; Liu et al. 2022, 2021; Song et al. 2011; H. Wu et al. 2017, 2016; C. Yang, Feng, et al. 2025; Zhang et al. 2017; Zhao et al. 2016). IFNT transactivates bovine LIF receptor in the endometrium to promote receptivity (B. Ma, Zhang, et al. 2024), but suppresses LIF mRNA expression in the blastocyst (Bao et al. 2014). IFNT can also enter the maternal circulation and affect granulosa cells to promote corpus luteum survival, as well as exerting effects on other cells (Basavaraja et al. 2019; Oliveira et al. 2008; Talukder et al. 2023). Embryo sex affects IFNT expression (Schanzenbach et al. 2019), but sexual dimorphism can also emerge in response to maternal factors, such as colony stimulating factor 2 (CSF2) (Dobbs et al. 2014). Dose‐sensitive responses to other hormones can modulate IFNT production (Z. Wang et al. 2013). Interestingly, interferon stimulated gene 15 (ISG15) may regulate IFNT expression in the embryo and positively affect embryo development (Zhao et al. 2017). Additionally, IFNT signaling can be compromised by heat stress, which may contribute to reduced pregnancy rates under heat stress conditions (Sakai et al. 2021). Although heat stress can activate IFNT in in vitro produced blastocysts (Hickman et al. 2013), exposure of oocytes and zygotes to heat stress inhibits IFNT production (Amaral et al. 2020), revealing complex responses of embryos to stress that can affect IFNT expression and pregnancy establishment. The complex features of IFNT biology may allow IFNT to be employed therapeutically to promote or sustain pregnancy establishment, as well as allowing IFNT or downstream IFNT target genes (interferon stimulated genes, ISGs) to be employed as biomarkers for measuring embryo quality, predicting embryo survival following embryo transfer, optimizing embryo culture conditions and other methodologies (Bott et al. 2010), or monitoring pregnancy status, all of which can provide significant economic benefits in the livestock and dairy industries (Bennett et al. 2026; Bishop et al. 2025; Bott et al. 2010; Ghanem et al. 2022; Hue et al. 2018; Matsuyama et al. 2012; Mori et al. 2015; Rodina et al. 2009; Strangstalien et al. 2024; Toji et al. 2017).

Pig blastocysts do not produce IFNT like ruminants. They instead produce estradiol to suppress PGF2α in maternal circulation, which otherwise would lead to luteolysis, thereby providing for maternal recognition of pregnancy (Bazer and Johnson 2014) (Figure 6). They also produce interferons gamma and delta, which induce multiple actions in the uterus to support pregnancy (Bazer and Johnson 2014; La Bonnardiere et al. 1994). Maternal recognition of pregnancy in pigs involves sequential actions of progesterone, estradiol, interleukin 1B2 (IL1B2), prostaglandin E2 (PGE2) and interferon G (IFNG) (Geisert et al. 2024).

Blastocyst communication with the uterine environment plays a key role in establishing pregnancy by suppressing immune system attack and eliciting uterine expression of factors to support the embryo. This includes inflammatory processes and TB‐immune cell interactions that mediate maternal tolerance and facilitate blastocyst invasion (Mor 2008; Park and Yang 2011; Rice and Chard 1998; Schumacher and Zenclussen 2019; Sehring et al. 2022; van Mourik et al. 2009). The mechanisms that regulate the maternal immune system within the uterus are extensive and complex. The numbers, types and phenotypes of maternal macrophages, dendritic cells, T‐regulatory cells, natural killer cells, neutrophils, and mast cells change during the reproductive cycle in response to hormones, in response to the initial encounter of the embryo with the endometrium, and in response to subsequent invading TB cells, with notable changes occurring at the site of embryo implantation that are crucial for successful implantation (Zenclussen and Hammerling, 2015). Dendritic cells, B cells, and T‐regulatory cells play crucial roles in the communication of the endometrium with the implanting blastocyst early during implantation and are required for this process (Teles et al. 2013; Zenclussen and Hammerling, 2015). An extensive body of literature describes cytokines and immune‐modulatory factors produced by invading cytotrophoblasts and syncitiotrophoblasts that regulate maternal immune system cells and factors that regulate spiral artery remodeling. These studies often employ placental samples or cultured cell lines and often describe TB functions and interactions with uterine cells that occur well after the initial interaction of the blastocyst with the endometrium. Less well characterized, particularly in humans, are the immune‐modulating factors produced by blastocysts that promote initial implantation. In cattle, thousands of genes are modulated in the uterus by the presence of an embryo as early as day 17 of gestation, an effect that includes modulation of the maternal immune system (Walker et al. 2010). In vitro exposure of bovine endometrial cells to day 8 blastocysts (but not earlier stage embryos) elicits significant changes in gene expression, including induction of ISGs (Passaro et al. 2018).

Some analyses have allowed discrimination between direct and indirect effects of the embryo. Human pre‐implanting blastocysts express a range of pro‐inflammatory and anti‐inflammatory cytokines (Seshagiri et al. 2016; Vani et al. 2023). Comparisons of samples of human embryo spent culture media for pregnant or non‐pregnant outcomes revealed that embryos produced multiple cytokines and growth factors that were able to modulate dendritic cell functions, which in turn affect T‐regulatory cell functions (Kyvelidou et al. 2024). In another study, human blastocyst culture medium analysis identified interleukin 18 as one secreted factor with potential value for quantifying embryo quality (Yi et al. 2025). Preimplantation stage embryos and blastocysts express in a species‐dependent manner interferon‐γ and type 1 interferon, and these factors can play critical roles in pregnancy, including vascular remodeling, angiogenesis, and decidualization (Murphy et al. 2009). Human endometrial cells cultured with or without blastocysts display many genes altered by interaction with the blastocyst (Han et al. 2021). Transcriptome analyses of bovine uterine epithelial cells with and without blastocysts, and of in vivo versus in vitro developed blastocysts revealed overlapping pathway regulators as possible mediators of embryo‐uterine dialogue (Noguchi et al. 2022). Other studies of uterine endometrial cells cultured with embryos, and with or without direct contact, revealed embryo‐induced changes in endometrial cell gene expression (Sponchiado et al. 2020). Single‐cell analyses of different types of porcine endometrial and TB cells further revealed gene expression differences related to direct embryo‐endometrium interactions, such as embryonic retinol binding protein 4 (RBP4) interacting with the stromal cell signaling receptor and transporter of retinol (STRA6) (Zang et al. 2024). Single‐cell transcriptomics of mouse embryo implantation sites allowed blastocyst signals to be inferred (Yang et al. 2021). These latter‐mentioned studies in humans, mice, pigs and cattle have thus revealed some of the mediators of direct interactions between embryo and uterus during the early periods of interaction, laying a foundation for additional mechanistic studies.

### 5.3 Metabolites

Metabolic changes in the blastocyst can lead to the production and release of metabolites that participate in communication with the uterine environment, including amino acids, succinate, acetyl CoA, and lactic acid. These metabolites can interact with target cells by acting as extracellular signals, altering G protein coupled receptor signaling, and affecting epigenetic DNA and histone modifications (Gurner and Gardner 2025).

Lactate is produced and released by the blastocyst (Gurner and Gardner 2025). About half of the glucose used for blastocyst metabolism is converted to lactate, which can serve metabolic roles in the embryonic cells, including coping with an anaerobic environment at the implantation site (Gurner and Gardner 2025). Taking into account the biology of cancer cell invasion and wound healing, as well as observations in the uterus, lactate release at the implantation site may lower local pH and promote uterine receptivity, immune tolerance, angiogenesis, and TB invasion (Gardner 2015; Gurner et al. 2022; Gurner and Gardner 2025). With respect to signaling mechanisms, lactate can activate the G‐protein coupled receptor GPR81, which can in turn modulate other pathways to promote cell survival and growth. Lactate may also activate GPR132 directly or indirectly to modulate other cellular phenotypes (Gurner and Gardner 2025). Lactate may promote blastocyst expression of MMPs, stabilize hypoxia inducing factor 1 subunit alpha (HIF1A) and promote expression of VEGFA to increase vascular permeability and angiogenesis and thereby facilitate implantation and pregnancy establishment (Gardner 2015; Halder et al. 2000). Lactate release may also promote post‐translational modification (lactylation) of histones (Gurner and Gardner 2025). In ruminants, fructose production in the uterus may supply essential metabolic demands of the embryo and increase lactate production to facilitate placentation (Bazer et al. 2025).

At later stages, placental metabolites such as amino acids (e.g., l‐arginine) and methylenetetrahydrofolate dehydrogenase 2 (MTHFD2) further promote or modulate TB invasion or support conceptus metabolic needs and elongation (Bazer et al. 2025; Gao et al. 2025; Q. Xu, Zhou, et al. 2025). The MTOR‐mediated nutrient‐sensing pathway is activated in TE cells by glucose, fructose, and amino acids including arginine in sheep (Johnson et al. 2018). Arginine affects other pathways as well (e.g., nitric oxide signaling), promotes TE cell proliferation and cellular remodeling, can enhance embryo survival in multiple species, and can promote integrin expression to support implantation (Johnson et al. 2018; Nakazato et al. 2024; Paudel et al. 2025). Other studies reveal effects of the embryo on uterine metabolism and luminal metabolites and effects of maternal metabolites on decidualization and implantation (Mancini et al. 2021; Sponchiado et al. 2019; Yang et al. 2024, 2022). Low succinate levels and elevated succinate dehydrogenase expression due to succinate dehydrogenase complex iron sulfur subunit B (SDHB) gene DNA hypomethylation in the chorionic villus cells are associated with pregnancy loss during the first trimester (X. H. Wang et al. 2021). Succinate and malate supplementation in vitro enhance hamster embryo viability, but not bovine embryo viability (Ain and Seshagiri 1997; Detraux and Renard 2022). Succinate also inhibits histone and TET DNA demethylases and inhibits certain dioxygenase enzymes, thereby affecting epigenetic cellular programming, and preserves pluripotency in embryos (Detraux and Renard 2022). These observations indicate the existence of a complex and species‐dependent system of metabolic communication between embryo and uterus, wherein metabolites affect metabolism, key signaling processes, epigenetic processes, proliferation, differentiation, implantation, and survival of the embryo.

### 5.4 Secreted Proteome Studies

Recent studies have provided systematic analyses of blastocyst secreted proteins, demonstrating a rich array of proteins in addition to those mentioned above. These secreted proteins may provide for additional communication between the embryo and uterus. Potential biomarker protein studies as well as protein profiles of spent culture media from blastocysts or blastocyst‐derived TB cells have been performed in diverse species, including mouse, cat, human, cattle, and horse (Arianmanesh et al. 2025; Chen et al. 2021; Dominguez et al. 2008; Freis et al. 2021; Katz‐Jaffe et al. 2006; Mathew et al. 2022; McReynolds et al. 2011; Raes et al. 2023; Swegen et al. 2017; Toporcerova et al. 2025). A proteomic analysis of secreted proteins in spent embryo culture media from mouse and human blastocysts identified several proteins as being more highly secreted by blastocysts as compared to earlier stages in mouse embryos, and being more highly secreted by viable human blastocysts as compared to degenerated embryos (Katz‐Jaffe et al. 2006). This analysis identified ubiquitin as a differentially expressed secreted marker of embryo viability (Katz‐Jaffe et al. 2006). Other studies identified proteins that were more highly secreted or depleted in culture medium comparing blastocysts that subsequently implanted to those that failed to implant (Dominguez et al. 2008; Freis et al. 2021), comparing arrested to developing embryos, comparing fast to slow developing embryos (Lindgren et al. 2018), or comparing aneuploid to euploid embryos (McReynolds et al. 2011). In cattle, comparisons of proteome profiles of uterine luminal fluid within individual animals but with and without blastocysts present identified differentially expressed proteins, with subsequent studies identifying secreted proteins expressed by embryos (Muñoz et al. 2012). Bovine cathepsin‐L was identified as an embryotrophic factor produced by higher quality blastocysts (Raes et al. 2023). Studies of soluble proteins analyzed separately from extracellular vesicle proteins identified hundreds of soluble proteins secreted by bovine primary TE cells, including ECM proteins, growth factors, cytokines and other putative regulators of epithelial‐mesenchymal transition and immunomodulators that may impact uterine cells and underlie observed chemotactic effects on endometrial mesenchymal stem cells (Calle et al. 2021). Proteomic analyses of whole cat blastocysts cultured with or without zonae pellucidae followed by spent culture medium analysis led to the identification of effects of zona removal on secreted proteins (Veraguas‐Davila et al. 2024). A study of equine embryos identified proteins of interest expressed in blastocoel fluid as well as secreted proteins (Swegen et al. 2017). Studies of secreted proteins and other factors provide insight into embryo physiology, mechanisms of embryo‐maternal communication, and potential markers for use in assessing embryo quality.

### 5.5 Extracellular mRNAs, miRNAs and Long Non‐Coding RNAs

Embryos express a wide variety of RNA molecules including mRNAs and small and long non‐coding RNAs (Russell et al. 2024). RNA molecules have been identified in blastocoel fluid, embryo culture medium, and TB cell culture medium. Using bioinformatic predictions of mRNA targets, these non‐coding RNAs have been associated with potential regulation of cellular processes in a range of cell types within the uterus as well as autocrine effects within the blastocyst, such as regulating ICM pluripotency and other processes (Badovská et al. 2025; Battaglia et al. 2025; Caponnetto et al. 2025; Chen et al. 2025; Coenen et al. 2024; Esmaeilivand et al. 2022; Fan et al. 2024; Guan et al. 2025; Nabeel and Nowak 2026; Omes et al. 2024; Pan et al. 2025; Potiris et al. 2024; Rutigliano et al. 2025; Saadeldin, Pavani, et al. 2025; Sun et al. 2026; Townsend et al. 2025; X. Wang, Cai, et al. 2025). These RNA molecules include those contained in extracellular particles (see below), and encompass microRNAs, mRNAs, circular RNAs, and long non‐coding RNAs. Effects of these secreted RNAs on implantation and pregnancy can be either positive or negative, depending on expression level.

## 6 Blastocyst‐Derived Extracellular Vesicles and Particles (EVPs)

**Figure 7:**
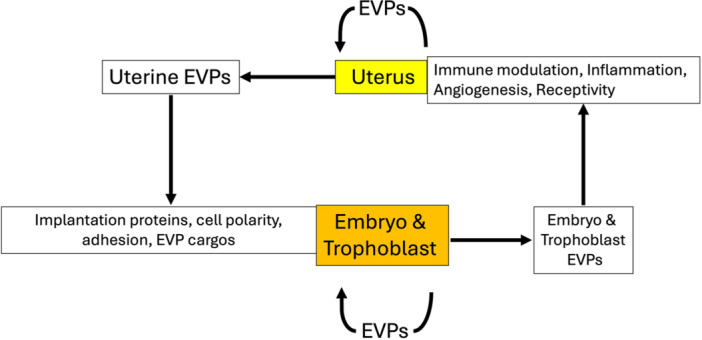
Summary of roles of extracellular vesicles and particles (EVPs) in pregnancy establishment. Uterine EVPs mediate essential communication between uterine compartments and are released into the uterine lumen where they can elicit changes in the embryo, including modifications of embryonic EVP cargos. Embryonic and TB EVPs exert autocrine effects as well as eliciting changes within the uterus.

In addition to secreted soluble proteins and other secreted factors, a remarkable discovery in recent years has been that EVPs produced by blastocysts and by uterine cells provide a complex and novel bidirectional system for embryo‐maternal communication (Figure 7). Different types of EVPs are produced by different cellular mechanisms, are of different sizes, and can have diverse cargo molecules. These include lipid bilayer membrane‐enclosed small and large extracellular vesicles (exosomes, ectosomes, apoptotic EVs) and small non‐vesicular extracellular particles (NVEPs) not enclosed with lipid bilayers and either having a lipid monolayer or no lipid coat (exomeres and supermeres) (Jeppesen et al. 2024; Shao et al. 2018; L. Yu, Shi, et al. 2025). The methods for extraction, isolation, purification and analysis of EVPs have continued to evolve and provide new biological insights with the discovery of molecular components that distinguish EVPs derived from different cells and tissues, and distinguishing those obtained under different physiological states. EVPs provide novel inter‐cellular signaling mechanisms (Meldolesi 2018), with distinct organ distributions, and a potential for selective purification and/or selective targeting to cells by virtue of specialized EVP components (L. Yu, Shi, et al. 2025; Zhang et al. 2018). EVPs may thus contribute to communication between cells, between organs, and possibly between embryos and uterus. EVPs have emerged as promising entities for basic research to study cellular regulation and for discovering novel biomarkers for disease and disease monitoring. Due to their ability to cross from circulation to target cells and organs, cross the placenta, and even cross the blood‐brain barrier, EVPs may allow development of novel, targeted delivery vehicles for drugs or small RNAs for treating diseases (Dieu et al. 2025; Dinc and Ardic 2026; Lewis et al. 2025; L. Yu, Shi, et al. 2025), which might include delivery of molecules to uterus, embryos, placentae or developing fetuses.

Many studies have revealed roles for EVPs in implantation and pregnancy. EVPs may be produced by the embryo, TB cells and cell lines, and cells in the uterus, and play significant roles in embryo‐maternal communication, participating in a complex dialogue (Fazeli and Godakumara 2024; Su et al. 2022). EVP cargos vary with embryo origin, quality and developmental stage (Gutierrez‐Reinoso et al. 2026). Decidual EVP cargos are sensitive to maternal hormonal regulation (H. M. Wu, Chen, et al. 2022). EVPs from endometrial cells, decidual cells, embryos, and TB cells can contain diverse arrays of proteins, microRNAs, small and long non‐coding RNAs, and lipids (Bang et al. 2025; Nabeel and Nowak 2026; Noguchi et al. 2024; Saadeldin, Pavani, et al. 2025; Townsend et al. 2025; H. M. Wu, Chen, et al. 2022). Uterine luminal fluid contains EVPs released by both the uterus and the conceptus, with similarly diverse arrays of cargos (Beal et al. 2023; Szuszkiewicz et al. 2022). These EVP components promote embryo adhesion, exert immunomodulatory effects, promote endometrial receptivity and maternal tolerance, regulate decidual cell apoptosis, and contribute to successful embryo attachment, apposition, invasion, and pregnancy establishment (Beal et al. 2023; H. M. Wu, Chen, et al. 2022). Embryo‐derived EVPs studied in multiple species modulate functions in the uterus as well as exerting beneficial autocrine effects within the embryo (Nabeel and Nowak 2026; Rutigliano et al. 2025; Saadeldin, Pavani, et al. 2025; Townsend et al. 2025). Bovine primary TE cells release EVPs that contain over 1200 proteins including ECM proteins, cell adhesion proteins, growth factors, protease inhibitors, and other proteins affecting multiple other cellular processes, such as epithelial‐mesenchymal transition, pregnancy and angiogenesis (Calle et al. 2021). These bovine EVPs also provide chemotactic signals to endometrial and peripheral mesenchymal stem cells (Calle et al. 2021). Conversely, EVPs released from these maternal cells affect the expression of implantation proteins in the TE cell EVPs (Calle et al. 2021). Thus, the uterine EVPs regulate TE cell functions, and TE and TB cells produce EVPs that modulate cell functions within the uterus (H. M. Wu, Chen, et al. 2022; Yan et al. 2025). Bovine uterine fluid EVP cargos and the cargos of EVPs released in vitro by endometrial cells are sensitive to the presence/absence of a conceptus, with observed differences being related to inflammation, immune modulation, endometrial receptivity, embryo cell polarity, cell adhesion, stem cell differentiation, and IFNT signaling (Mazzarella et al. 2025). Trophoblast‐derived EVPs can also modulate cell senescence (Abdelmohsen et al. 2026). Embryo‐derived EVPs and uterine EVPs also vary with embryo quality, developmental stage, uterine receptivity, and maternal health, suggesting possible value of EVP components as valuable biomarkers for diverse clinical applications (Gutierrez‐Reinoso et al. 2026; Nabeel and Nowak 2026).

## 7 Maternal Health and Environmental Factors Impacting Embryonic Signaling

With the complexity of the embryo‐maternal dialogue and the complexity of the signals produced by blastocysts and TB cells, there are many aspects of maternal health and environment that can adversely affect embryo implantation and pregnancy establishment. Maternal health effects on implantation are extensive. Embryo exposures can also impact implantation success.

Maternal mutations comprise one source of factors that can affect implantation. These can include factors that affect oocyte quality, embryo ploidy, embryo viability, and embryo dialogue with the uterus (Amano et al. 2016; Chen et al. 2026; Enciso et al. 2016; Hassan et al. 2025; Králíčková et al. 2005; Pan et al. 2019; Takezawa et al. 2023; Yalcin et al. 2025), as well as polymorphisms, mutations or chromosomal abnormalities affecting the maternal immune system, uterine angiogenesis, and endometrial function (Jalilvand et al. 2022; Králíčková et al. 2005; Moustakli et al. 2025; Yaron et al. 1996).

Maternal age affects implantation rates and pregnancy outcomes independent of embryo aneuploidy (Reig et al. 2020; Vitagliano et al. 2023). However, understanding the basis for this effect may require further study. A recent meta‐analysis of outcomes in patients receiving treatments using assisted reproduction technologies (ARTs) with euploid embryo transfer revealed significantly greater pregnancy, live birth and implantation rates for women below versus above age 35 (Vitagliano et al. 2023). Similar results were seen comparing women below age 35 with those above 38 and comparing women below 38 versus those above 38 (Vitagliano et al. 2023). Maternal age can affect TE non‐coding RNAs and implantation (Ntostis et al. 2023). Factors contributing to the maternal age effect on implantation and pregnancy rate could include age‐dependent changes in endometrial gene expression, function and receptivity (Devesa‐Peiro et al. 2022; Marti‐Garcia et al. 2024; Ntostis et al. 2023; Pathare et al. 2023; Zhao et al. 2023), non‐ploidy related changes in embryo quality (Ntostis et al. 2023), or other health or disease conditions that may vary with age (Vitagliano et al. 2023). However, one study reported that the rate of in vitro blastocyst formation for euploid embryos was not affected by maternal age (Irani et al. 2019) and another study reported that implantation and miscarriage rates for euploid human embryos were not associated with embryo morphology (Viñals Gonzalez et al. 2019), arguing for non‐embryo factors being responsible for the maternal age effect on implantation rate and pregnancy outcome.

In addition to age‐related changes in the endometrium, other endometrial factors impact implantation and pregnancy rates. Endometriosis and adenomyosis are associated with infertility and pregnancy complications and with defects in decidualization and endometrial immune cell regulation (Shi et al. 2025). One recent analysis identified a discrete set of aberrantly regulated genes in common for alterations in endometrial gene expression profiles for patients with recurrent implantation failure and endometriosis, including a group of five genes (SRP Receptor Subunit beta, SRPRB; RNA binding motif protein 3, RBM3; insulin induced gene 2, INSIG2; glycogenin 1, GYG1; and F‐Box and WD repeat domain containing 2, FBXW2) that were associated with an inflammatory microenvironment and down‐regulated in both conditions (J. Yu, Wang, et al. 2025). The functions for these proteins include roles in protein secretion, mRNA regulation, cholesterol and lipid metabolism, protein turnover, and immune regulation (J. Yu, Wang, et al. 2025). Dysregulation of the immune system within the endometrium, including dysregulation of regulatory T‐cell function and macrophages, is associated with endometriosis (Marečková et al. 2024; Taguchi and Hayashi 2025), and may contribute to an inflammatory environment, lack of receptivity and reduced immune tolerance, and toxic effects in the embryo, thereby diminishing successful implantation and placentation (Baharaghdam et al. 2026; Robertson et al. 2018; Shi et al. 2025; Taguchi and Hayashi 2025). A key player in endometrial regulation, uterine receptivity, decidualization and implantation is Homeobox A10 (HOXA10) (Mishra and Modi 2025). This key gene is essential for implantation (Bagot et al. 2000) and is regulated in the adult uterus by steroid hormones, vitamin D and environmental factors at transcriptional, post‐transcriptional and post‐translational levels (Caserta et al. 2021; Mishra and Modi 2025). It can be downregulated by genetic mutation, epigenetic regulation, and exposures to environmental toxins, and is down‐regulated in women with recurrent implantation failure, adenomyosis and other uterine pathologies (Caserta et al. 2021; Mishra and Modi 2025).

Maternal diet can also impact the embryo and the uterus and thereby impact implantation and placentation. Diverse vitamins, nutrients and other components may affect embryo implantation via effects on the uterus, on the embryo or on the embryo–uterus dialogue (Dahlen et al. 2022; Drzewiecka et al. 2021; Hoek et al. 2020; Kalisch‐Smith et al. 2019; Ma et al. 2022; Zglejc‐Waszak et al. 2019).

Vitamin D can affect signaling through HOXA10 and thereby affect embryo attachment, uterine receptivity, immune cell function, and implantation (Pîrlog et al. 2025). Vitamin D deficiency in mice impairs decidualization and down‐regulates expression of WNT4 (Yi et al. 2026). Vitamin D deficiency can negatively impact pregnancy outcome, particularly in women with recurrent implantation failure, and supplementation may be one component in a plan for improving implantation and outcome (Badihi et al. 2025; Chen et al. 2020; Kuroda 2024; K. Wang et al. 2024). However, some studies raised questions about the benefits of vitamin D supplementation in women undergoing in vitro fertilization (IVF) or indicated that vitamin D level in follicular fluid may be more predictive of outcome than serum level (Cai et al. 2021; Muyayalo et al. 2022). Some of the differences in results may reflect a complex system wherein optimum levels of vitamin D combined with correct regulation of its receptor work together to impose key control over cellular differentiation and decidualization. A recent study found that Vitamin D receptor gene expression decreases during decidualization in a transformed human endometrial stromal cell line used as a model of decidualization; importantly, receptor knockdown promoted decidualization but over‐expression inhibited it in this cell model, with extensive effects on gene regulation and chromatin accessibility that are sensitive to vitamin D and that involve other chromatin regulators (Yi et al. 2026). These observations may improve approaches to interventions in infertility related to vitamin D.

Other dietary effects on implantation have been reported. Dietary selenium, but not selenium excess, can enhance embryo quality and implantation (Dahlen et al. 2022). Soluble fiber (inulin) may modulate pig uterine exosomes, alter endocrine profile, promote angiogenesis, promote embryo development, and thereby promote implantation (Chang et al. 2025). Whole grain intake can enhance pregnancy outcome via effects on endometrial thickness and receptivity (Gaskins et al. 2016). Dietary chenodeoxycholic acid can affect steroidogenesis and promote embryogenesis (Chen et al. 2024). Maternal ketone β‐hydroxybutyrate consumption can negatively affect preimplantation embryo development and implantation rate (Whatley et al. 2022). High dietary fat and specific lipid composition may affect embryo elongation in cattle and may adversely affect decidualization in other species (Z. Chen, Yiwen, et al. 2023; Giller et al. 2018). Interestingly, a high fat diet may counteract effects of some toxin exposures (Martinez et al. 2015). Lipid metabolism and availability and composition of serum free fatty acids can exert a complex and diverse range of positive or negative effects on the endometrium by modulating inflammation, steroidogenesis and multiple signaling processes, so that dysregulation of lipid metabolism and dietary fat intake can affect uterine receptivity (Yang et al. 2022). Maternal diet can also affect embryo epigenetic programming, and immediate and long‐term embryo quality and development (Denisenko et al. 2016; Fleming et al. 2017; Fleming et al. 2011; Zglejc‐Waszak et al. 2019). Thus, maternal diet can impact both sides of the embryo‐uterine dialogue by altering embryonic gene expression and by altering the uterine environment.

Maternal diabetes, obesity, and increased adiposity can also impact embryo implantation. Diabetes can diminish successful pregnancy by impairing decidualization, ECM remodeling, TB invasion, spiral artery remodeling, and placenta development. These deficiencies may be associated with inadequate maternal immune tolerance as well as maternal endocrine and metabolic disruptions, and excessively elevated estrogen levels that inhibit LIF expression and LIF‐STAT3 signaling (Favaro et al. 2013; Groen et al. 2015; Walewska et al. 2025; T. S. Wang, Gao, et al. 2015). Obesity diminishes fertility in diverse species by altering endometrial gene expression. Among different studies in different species, these changes variably included negative effects on embryo implantation [increased expression of genes related to immune response, increased pro‐inflammatory genes, reduced anti‐inflammatory genes, increased inflammation, increased apoptosis, increased lipid deposition, increased reactive oxygen species production, and increased leptin signaling] as well as accelerated or enhanced angiogenesis that may increase blood flow leading to fetal overgrowth (Gonnella et al. 2024; Walewska et al. 2025, 2024). Deficiencies in uterine EVPs are also associated with obesity and may exert negative effects on receptivity (Galio et al. 2023). Importantly, obesity alters the uterine environment before pregnancy, including insulin resistance, increased cell proliferation, uterine hypoxia, and changes that delay embryo transport through the oviduct, alter embryo positioning within the uterus, and modify uterine contractility (Bazzano et al. 2021, 2018). These changes may compromise the synchrony needed for embryos to interact with a receptive endometrium during the window of implantation (Bazzano et al. 2021). Changes in global DNA methylation and DNA methyltransferase expression in the endometrium with obesity may contribute to changes in gene expression (Bozdemir et al. 2024). Interestingly, the effects of maternal obesity on IVF outcomes may be more prevalent in older women (Liu et al. 2023). In addition to effects in the uterus, elevated exposures of preimplantation embryos to maternally supplied lipids can lead to low birth weight and catch‐up growth postnatally (Jungheim et al. 2011), and changes in serum lipid profiles may compromise oocyte quality, with later effects in the embryo (Ruebel et al. 2022). Thus, as with maternal diet, diabetes, obesity and adiposity may affect implantation success via effects on both the embryo and the uterus.

Microbes can also affect implantation, encompassing positive effects of a healthy microbiome and negative effects of infections leading to inflammation. Numerous changes in the endometrial microbiome are associated with different gynecological diseases and may affect uterine receptivity and implantation (Blazheva et al. 2024; Castellanos‐Ruiz et al. 2025; Chen et al. 2022). Positive and negative associations of specific vaginal and uterine microbes on pregnancy have been reported (Alley et al. 2025; Motiwala et al. 2026; Zhao et al. 2025). Complex interactions between hormone treatments, the immune system, the microbiome, and uterine receptivity and immune tolerance set the stage for microbial effects on implantation and pregnancy (Motiwala et al. 2026). Treatment of sows with certain microbial metabolites can positively affect embryo adhesion (Ye et al. 2025). Microbial profiling and interventions targeting the microbiome may offer new approaches to improve implantation and fertility in some situations (Doroftei et al. 2026; Kadogami et al. 2020; Stoyancheva et al. 2025). Pathogenic bacteria in the endometrium are also associated with negative effects on implantation (Zou et al. 2023). Strikingly, the gut microbiome can also affect the uterus and implantation success by modulating the immune system systemically (Rao et al. 2026). It has also been suggested that periodontitis may create a systemic inflammatory condition that could contribute to diverse conditions including endometriosis and effects on implantation (Machado et al. 2020), and this is something that may be addressed clinically.

A variety of other maternal environmental factors, such as maternal stress (Breton‐Larrivée et al. 2019; Burkuš et al. 2015; Lian et al. 2022; Mousavi et al. 2024; J. Wu, Lin, et al. 2022; J. X. Wu et al. 2021), maternal exposure to cannabis (Correa et al. 2016; Ezechukwu et al. 2020; Lo et al. 2022), and exposure to other environmental agents (Breton‐Larrivee et al. 2019; Kalisch‐Smith et al. 2019; Pérez‐Tito et al. 2014; G. Yang, Yao, et al. 2025) are associated with effects on implantation. These exposures may affect early embryogenesis and implantation through endocrine effects, epigenetic changes, endometrial changes, effects on embryo physiology and cell lineages, and effects on crucial signaling pathways. Exposure to endocrine disruptors and other pollutants can also impact implantation by disrupting the regulation of expression of hormone receptors and other regulators of endometrial receptivity, decidualization, immune tolerance, and possibly the endometrial microbiome (Caserta et al. 2021; Castellanos‐Ruiz et al. 2025; Hwang et al. 2026; Karwacka et al. 2019; Schjenken et al. 2021; Zhang et al. 2025). Reducing harmful maternal stress and environmental factors could prove beneficial in clinical and agricultural applications to enhance reproductive outcomes.

## 8 Novel Diagnostics and Therapeutic Interventions Related to Embryo Implantation

**Figure 8:**
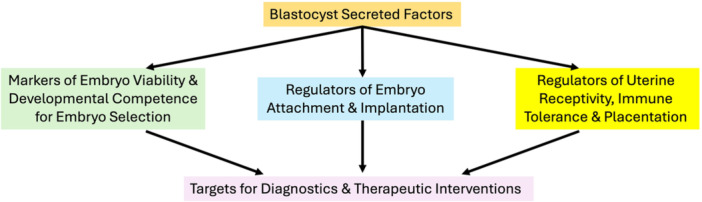
Relationship of embryo‐expressed factors to three aspects of implantation biology, and their potential uses in applied and clinical reproduction.

**Figure 9:**
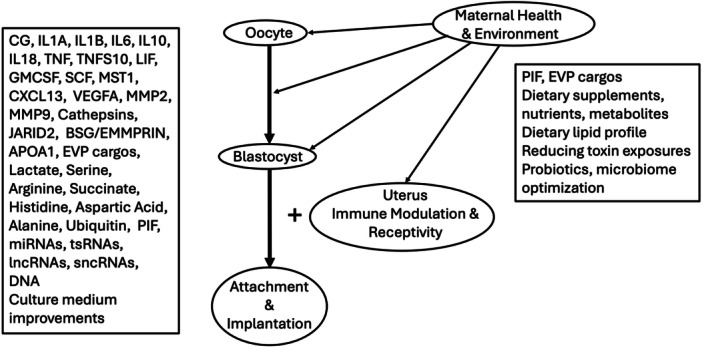
Examples of potential targets for novel approaches in applied reproduction and clinical interventions to enhance ART outcomes or provide for contraception. Maternal health and environmental factors can impact multiple steps in the development from oocyte to blastocyst and subsequent implantation, as well as impacting uterine recognition of pregnancy, immune tolerance, and receptivity. Embryo‐expressed factors as possible targets for intervention, such as positive or negative embryo selection or embryo improvement are in the box at left. Maternal factors that may be useful for enhancing uterine receptivity are in the box at right. Further testing of such possibilities in humans or for application in other individual animal species are needed. APOA1, apolipoprotein A1; BSG/EMMPRIN, Basigin; CXCL3, C‐X‐C motif chemokine ligand 3; CG, chorionic gonadotropin; EVP, extracellular vesicles and particles; GMCSF, granulocyte macrophage colony stimulating factor; IL10, interleukin 10; IL18, interleukin 18; IL1A, interleukin 1α; IL1B, interleukin 1β; IL6, interleukin 6; JARID2, Jumonji and AT‐rich interaction domain containing 2; LIF, leukemia inhibitory factor; lncRNA, long non‐coding RNA; miRNA, microRNA; MMP2 and 9, matrix metalloproteinase 2 and 9; MST1, macrophage stimulating 1; PIF, preimplantation factor; SCF, stem cell factor; sncRNA, small noncoding RNA; TNF1, tumor necrosis factor; TNFS10, TRAIL or TNF‐related apoptosis‐inducing ligand; tsRNA, tRNA‐derived small RNA; VEGFA, vascular endothelial growth factor A.

The dramatic progress in better understanding the mechanisms that enable successful embryo implantation and pregnancy establishment has led to the continuing emergence of new approaches to diagnose the causes of infertility related to embryo implantation and the embryo‐uterine dialogue (e.g., recurrent implantation failure, recurrent pregnancy loss, other disorders), as well as approaches to enhance pregnancy success, or novel approaches for contraception. These novel diagnostic and therapeutic approaches can be generally grouped into three categories related directly to the factors produced by the embryo (Figures 8 and 9). These approaches may offer new opportunities for enhancing ART success in livestock and endangered species, as well as possible new approaches in clinical human reproduction methods.

As discussed above, embryos express numerous factors that mediate effective communication with the uterus and thereby facilitate implantation and pregnancy establishment. Embryos also release a wide variety of macromolecules into culture medium, including nuclear and mitochondrial DNA, metabolites, proteins including growth factors and cytokines, CG, small and large RNA molecules, and EVP cargos, and these have been studied as potential biomarkers of embryo quality (Butler et al. 2013; Saadeldin, Pavani, et al. 2025; Seli et al. 2007; Toporcerova et al. 2025; Zmuidinaite et al. 2021). Many embryo‐expressed factors can thus be considered as candidates for selecting the most implantation‐competent embryos, modulating embryo implantation potential, enhancing uterine receptivity, or as novel targets for contraception (Figures 8 and 9). Several components bear specific mention here. Apolipoprotein has been studied in embryo culture medium as a potential biomarker, but with discrepant conclusions, possibly related to dynamic regulation in blastocysts that creates challenges for accurate timing of analyses to account for differences in developmental rate between euploid and aneuploid embryos (Arianmanesh et al. 2025; Toporcerova et al. 2025). Embryo‐secreted cytokines such as interleukin‐18 (IL18), TNF superfamily 10 (TRAIL/TNFSF10), LIF, IL6 and colony stimulating factor 2 (GMCSF/CSF2) in embryo culture media have also been suggested as useful biomarkers in selecting embryos for transfer that may have higher implantation potential (Chen et al. 2021; Z. Li, Li, et al. 2020; Yi et al. 2025; Zhong et al. 2020). Preimplantation factor (PIF) produced by the embryo can induce a more favorable decidual environment for implantation through its effects on human endometrial epithelial cells (Barnea et al. 2012). Metabolites have also been suggested as markers for embryo selection, and comprise another emerging area (Seli et al. 2007; Toporcerova et al. 2025). Components of embryo‐derived EVPs have emerged as additional markers of embryo quality that may be applicable to IVF and embryo transfer programs (Saadeldin, Pavani, et al. 2025). In one study of bovine embryo‐produced EVPs, in vitro derived embryos yielded stronger effects on endometrial cell gene expression than in vivo produced embryos, indicating that EVP content varies with embryo origin or quality, and that EVPs can be used to manipulate endometrial cell phenotype (Aguilera et al. 2024). Moreover, devising bioengineered EVPs (Cai et al. 2025; Ebrahimi et al. 2025; Obuchi et al. 2025) for loading and delivery of specific cargos to the embryo or to the endometrium could provide new approaches for the delivery of specific molecules (miRNAs, mRNAs, proteins, etc.) that could promote improved embryo implantation. Additionally, synthetic forms of embryo‐expressed gene products (e.g., PIF) might be applicable for eliciting a uterine environment that is more favorable for implantation (Barnea et al. 2012), although this capacity may vary with species (Wonfor et al. 2020). Further study of the pathways and embryo‐secreted factors described above could yield additional novel markers for identifying embryos with the greatest implantation potential and the greatest ability to signal to the uterus to promote a receptive environment, and for developing methods to enhance these embryo properties. Interference with embryo apposition, attachment, invasion, or embryo‐uterine dialogue also could provide novel approaches to contraception.

Embryo exposures, such as culture effects during ART, as well as embryo selection methods can affect implantation success. Just as factors in embryo culture medium may prove useful for selecting embryos with greater implantation potential, modifying culture systems or in vitro embryo treatments to enhance implantation‐favorable gene expression profiles in the embryo may offer other avenues for enhancing ART outcomes. Pregnancy outcomes might be enhanced by using single step culture systems as compared to two‐step systems, possibly by reducing stress to the embryos that may arise during the change in medium (Máté and Török 2020). Culture medium variations can affect blastocyst transcriptome profiles (Ferreux et al. 2026), setting the stage for a possible systematic optimization of embryo culture conditions for maximizing not only lineage forming ability but also for achieving a pro‐implantation gene expression profile.

Addressing maternal health and environmental factors also may become more feasible as a means for enhancing fertility and pregnancy outcomes. Genetic testing may prove useful for identifying genetic factors, the effects of which might be addressed. Discovering ways to intervene in the signaling pathways that connect diverse health and environmental factors (e.g., diabetes, obesity, diet, microbial, stress, chemicals) to deficiencies in implantation could allow the impacts of a myriad of such health and environmental factors to be better managed clinically for improved outcomes. Additionally, serum‐born biomarkers of uterine receptivity (S. Xu, Hu, et al. 2025) may also prove useful in ART.

## 9 Conclusions and Perspectives

The foregoing sections highlight how much we have learned about embryonic processes that promote embryo implantation but also how much more we have left to discover. Much information on TB cell interactions with the uterus have come from in vitro culture and testing in TB cell lines or analyses of early stage placenta, obtained well after initial embryo–uterus interaction. Discovery of cellular and molecular mechanisms driving embryo apposition, attachment, invasion, and survival remains an active and important area of study. While many maternal and environmental factors have been identified that affect these processes, the contribution of embryonic responses to those factors and attendant effects on overall outcomes remains poorly understood. The knowledge of embryo‐derived factors promoting pregnancy establishment, such as those embryo factors participating in interactions with maternal immune cells, is limited for some species. Single‐cell transcriptomics may guide further discovery in this area. There is a need to understand better how embryonic processes may compensate for changes in the endometrium to lessen the effects of environmental factors on implantation, or how they may work additively in leading to negative outcomes. Importantly, recent innovations in studies using in vitro three‐dimensional organoids are providing new insights into the details of embryo interactions with the endometrium and endometrial biology, and offer additional means of testing effects of environmental factors on implantation, their mechanisms of action, and possible ways to mitigate those effects (Ahmad et al. 2024; Ciprietti et al. 2025; Devkota et al. 2026; Fraser et al. 2021; Fujimura et al. 2025; Kleinová et al. 2024; Kwon et al. 2025; McCutcheon et al. 2025; Molè et al. 2026; Rawlings et al. 2021; Saadeldin, Alshehri, et al. 2025; Song et al. 2026; Tinning et al. 2025).

Recent advances have raised many important new questions for basic research, such as the roles that EVPs play in implantation and pregnancy establishment, and the mechanisms of these effects. The expanding knowledge of EVPs is particularly noteworthy for the many questions being raised. For example, what is the degree of heterogeneity of EVPs produced by the uterus and by the embryo? Are different types of uterine and TB cells differentially able to take up certain kinds of EVPs? Are the EVPs thus targeted to specific cell types or sub‐populations of cells or do they act broadly on all cells in the responding side? What are the genetic factors affecting EVP cargo composition and cellular responsiveness? What are the relative roles of EVP cargoes versus secreted proteins and RNAs? How do these different roles affect sensitivity and responses to environmental factors? Additional discoveries are likely in the areas of lipids, metabolites, single carbon metabolism, mitochondrial function and modulation, embryo‐secreted signals, and many other factors. A greater understanding of the many intricate interactions between embryo and uterus, and between uterine stroma, uterine epithelium and immune system cells, particularly at the site of implantation, is also likely to emerge. How the environment, maternal health and both maternal and embryonic genetic factors impact attachment, implantation and pregnancy establishment also remains an important area for both basic and applied research.

The foregoing sections also provide cause for optimism that continued advancement of our knowledge of embryo implantation and the mechanisms that determine its success or failure will open the door to many new applied and clinical applications in reproductive biology. As discussed above, many different interventions to enhance embryo implantation and pregnancy outcomes are theoretically feasible. A greater understanding of embryonic signaling pathways, the roles of EVPs, the uterine responses to these factors, and how these functions can be modulated could allow more focused and efficacious interventions to be devised. Further studies are needed, however, to determine which biomarkers or potential interventions are most efficacious for human reproduction applications, or for use in individual animal species. Some interventions may be somewhat simple and easy to implement, such as dietary supplements, antibiotics to address infections, or probiotics to optimize the microbiome. Other interventions will likely require more sophisticated technologies, ranging from methods for in vitro embryo selection, to genetic testing, to evaluation or modification of uterine receptivity, immune tolerance and spiral artery remodeling, to enhancing embryo implantation potential in vitro, or to developing and delivering engineered EVPs to the embryo or the uterus. Realistically, opportunities to apply such new technologies may be limited by cost, risk, time constraints, and ethical considerations, but further discovery and refinement could open the door to greater feasibility, applicability and affordability.

## Author Contributions

**Keith E. Latham:** conceptualization, funding acquisition, writing – original draft, visualization, writing – review and editing, investigation, supervision, project administration, resources.

## Ethics Statement

The author has nothing to report.

## Conflicts of Interest

The author declares no conflicts of interest.

## Data Availability

Data sharing not applicable to this article as no data sets were generated or analysed during the current study.
